# Commissural Misalignment Following Valve‐in‐Valve Transcatheter Aortic Valve Implantation

**DOI:** 10.1002/ccd.70460

**Published:** 2026-01-07

**Authors:** Matthias Raschpichler, Johannes Rotta Detto Loria, Vivek Patel, Thilo Noack, Karoline Nüsser, Oliver Dumpies, Dhairya Patel, Takashi Nagasaka, Prateek Madaan, Aakriti Gupta, Holger Thiele, Michael A. Borger, Raj Makkar, Mohamed Abdel‐Wahab

**Affiliations:** ^1^ University Hospital of Cardiac Surgery Heart Center Leipzig at Leipzig University Leipzig Germany; ^2^ Department of Internal Medicine/Cardiology Heart Center Leipzig at Leipzig University Leipzig Germany; ^3^ Cedars‐Sinai Smidt Heart Institute Cedars‐Sinai Medical Center Los Angeles California USA

**Keywords:** aortic stenosis, aortic valve replacement, commissural alignment, valve‐in‐valve

## Abstract

**Background:**

Data on commissural misalignment (CMA) during valve‐in‐valve transcatheter aortic valve implantation (ViV‐TAVI) for valve failure after surgical aortic valve replacement (SAVR) is scarce.

**Aims:**

To study the impact of CMA on clinical and hemodynamic outcomes following ViV‐TAVI for failed SAVR.

**Methods:**

Data of patients who underwent ViV‐TAVI for failed SAVR valves at two institutions (Heart Center Leipzig at Leipzig University, Leipzig, Germany; Cedars‐Sinai Smidt Heart Institute, Los Angeles, USA) were retrospectively collected and compared regarding the existence of CMA. Outcomes of interest included Valve Academic Research Consortium (VARC)‐3‐based clinical endpoints, Computed Tomography‐based hypoattenuated leaflet thickening (HALT), hemodynamic outcome, and mid‐term all‐cause mortality.

**Results:**

Of the 687 patients who underwent ViV‐TAVI, post‐procedural CT of sufficient quality to measure commissural alignment was available in 180 patients (47.8% females, mean age 78.0 years). Self‐expanding TAVI valves were used in 49.2% of the cases. CMA was found in 35 individuals (19.4%) and was associated with reduced VARC‐3‐based technical success (80% vs. 93%, *p* = 0.03), driven by increased rates of coronary obstruction (17.1% vs. 5.6%, *p* = 0.03). The incidence of HALT was 31.7%, without differences in patients with and without CMA. VARC‐3‐based device success was 62.9%, driven by an incidence of residual mean valve gradient (≥ 20 mmHg) of 35.4%, without differences between groups. Midterm survival after a mean of 747 days was 84.6% and comparable between groups.

**Conclusions:**

For patients undergoing ViV‐TAVI for failed SAVR, technical success is reduced for cases of CMA due to higher odds of coronary obstruction, without differences in mid‐term survival.

**Abbreviations**
CMA: commissural misalignment
CO: coronary obstruction
CT: computed tomography
HALT: hypoattenuated leaflet thickening
SAVR: surgical aortic valve replacement
VARC: Valve Academic Research Consortium
ViV‐TAVI: valve‐in‐vave transcatheter aortic valve implantation

## 1 Introduction

Valve‐in‐valve transcatheter aortic valve implantation (ViV‐TAVI) is an established treatment for bioprosthetic valve failure after surgical aortic valve replacement (SAVR) in patients at increased surgical risk [1, 2]. Compared to redo SAVR, ViV‐TAVI is associated with improved short‐term mortality and morbidity, while data on longer‐term outcome remains controversial [3, 4]. Relevant drawbacks of ViV‐TAVI include residual transvalvular gradients and increased incidence of patient‐prosthesis mismatch, clinical valve thrombosis, and coronary obstruction (CO) [5, 6, 7].

The term commissural alignment describes the rotational orientation of a transcatheter heart valve (THV) with respect to native valve commissures. An established definition of commissural misalignment (CMA) considers a difference in rotational orientation of 30 degrees or more as moderately or severely misaligned [8]. Recent studies have demonstrated a potential impact of CMA on hemodynamic outcome, and aspects such as valve thrombosis, coronary access, and valve re‐intervention have also been analyzed [9, 10, 11].

Although ViV‐TAVI is increasingly performed, little data exists on the impact of CMA on hemodynamic and clinical outcomes in these patients [12]. The goal of this study was to assess the impact of CMA on outcomes in patients undergoing ViV‐TAVI following SAVR.

## 2 Methods

### 2.1 Study Population

We analyzed baseline, computed tomography (CT), procedural, and mid‐term clinical data of patients that underwent ViV‐TAVI following SAVR at two institutions (Heart Center Leipzig at Leipzig University, Leipzig, Germany, and Cedars‐Sinai Smidt Heart Institute, Los Angeles, CA, USA), from June 2018 to February 2024, and from March 2015 to January 2023, respectively. Patients who received post‐ViV CT imaging of sufficient quality to evaluate CMA of the implanted THV were included in this retrospective study.

The study was approved by the Ethics Committee of the Medical Faculty of the University of Leipzig as part of the Leipzig TAVI Registry (registration no. 102/20‐ek, NCT05015452). Individuals included at Cedars‐Sinai Smidt Heart Institute were part of the RESOLVE (Assessment of TRanscathetEr and Surgical Aortic BiOprosthetic VaLVve Dysfunction with Multimodality Imaging and Its TrEatment with Anticoagulation) registry (NCT02318342). Data and statistical analysis can be shared upon reasonable request to the corresponding author.

### 2.2 CT Analysis

Commissural alignment was analyzed using pre‐ and post‐ViV CT as previously described [10]. CMA was defined as > 30° difference between commissures of the implanted SAVR and the post‐ViV THV neo‐commissures [11]. Hypoattenuated leaflet thickening (HALT) was analyzed as previously described [13, 14]. Coronary alignment was analyzed as previously reported [8]. Coronary eccentricity was defined as at least moderate eccentricity (i.e., a deviation from a centered position of the coronary ostia > 20°). All analyses were performed using 3Mensio version 9.0 (Pie Medical Imaging) and Syngo.Via (Siemens Healthineers).

### 2.3 Outcomes of Interest

The primary outcomes of interest were Valve Academic Research Consortium (VARC)‐3‐based technical and device success, as well as CO, defined as unplanned stenting during ViV‐TAVI or VARC‐3‐based type 5 myocardial infarction [15]. Secondary outcomes of interest included HALT and mid‐term all‐cause mortality. The primary outcomes of interest were assessed at discharge. The secondary outcomes of interest were assessed at the time of post‐ViV CT scanning and at a median of 747 days post‐procedure, respectively. Clinical outcomes were verified for individual events using angiographic data and hospital records.

### 2.4 Statistical Analysis

Normality of data was evaluated using Shapiro−Wilk test. Continuous variables of normal distribution were compared using student *t*‐test and are shown as mean ± standard deviation (SD). Continuous variables without normal distribution were compared using Wilcoxon rank‐sum or Kruskall−Wallis test and are shown as median ± interquartile range (IQR). Categorical variables were compared using Chi^2^ test and are shown as frequencies and percentages. Missing variables were examined for possible associations and patterns of missingness and subsequently treated as missing at random. Using baseline parameters as independent variables and the outcome of CO as the dependent variable within univariable binomial regression, we tested all variables with a *p* value of less than 0.1 using the Wald‐test as well as Pseudo‐R^2^ measures as criteria, and the Hosmer‐Lemeshow goodness of fit for model diagnostics. Mid‐term all‐cause mortality was estimated and depicted using the Kaplan−Meier algorithm. Statistical analysis was performed using base R functions (version 4.2.1) within RStudio (version 2024.09.1 + 394), as well as the following R packages: *tidyverse*, *compareGroups*, *ggthemes*, *ggsci*, *ggpubr*, *aod*, *pscl*, and *finalfit*. The content follows principles of reproducible research.

## 3 Results

### 3.1 Study Population

A total of 687 patients underwent ViV‐TAVI for failed SAVR during the study period at both institutions. Institutional differences in baseline characteristics are shown in Supporting Information S1: Table 1. Post‐ViV CT was available in 180 patients (26.2%, 47.8% females, mean age of 78.0 years). Median time between ViV‐TAVI and CT imaging was 5 days (IQR, 3; 33).

### 3.2 CMA

**Figure 1:**
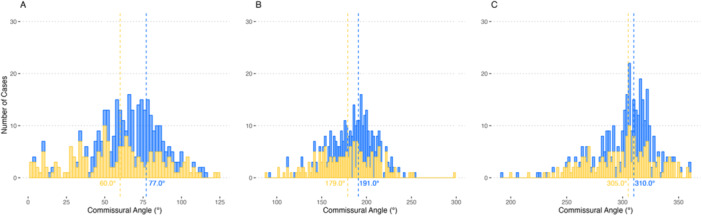
Distribution of prosthesis commissures relative to the right coronary ostium. (A) Distribution of commissures between the right and left coronary cusp. (B) Distribution of commissures between the left and noncoronary cusp. (C) Distribution of commissures between the noncoronary and right coronary cusp. The x‐axis shows commissural angles in degrees from the right coronary ostium. The y‐axis shows the number of cases. The vertical lines indicate the mean and median commissural angle for commissures of the previously implanted surgical aortic bioprostheses (blue) and neo‐commissures following valve‐in‐valve transcatheter aortic valve implantation (yellow), respectively. [Color figure can be viewed at wileyonlinelibrary.com]

CMA of the THV was detected in 35 individuals (19.4%) (Table 1). There were no differences in baseline clinical characteristics between groups. The median difference in alignment was 45.7 degrees (IQR, 38.0; 57.8) versus 5.0 degrees (IQR, −7.0; 23.8) for misaligned versus aligned THV (Figure 1). Of the deteriorated surgical prostheses, 91.6% were stented valves and 81 (49.4%) were prostheses at increased risk of CO (i.e., prostheses with externally mounted leaflets and stentless valves). Of the 35 cases of CMA, 22 (62.9%) were observed in these prostheses (*p* = 0.03). There were no differences regarding CT‐based annular or sinotubular measures between misaligned and aligned THV. Left main and right coronary eccentricity were present in 18.5% and 29.7%, respectively, without differences between groups (Table 2).

**Table 1:** Baseline characteristics.

	All *N* = 180	Aligned *N* = 145	Misaligned *N* = 35	*p* value
Age	78.0 [71.8; 83.0]	79.0 [72.0; 83.0]	75.0 [69.5; 80.5]	0.116
Females	86 (47.8%)	69 (47.6%)	17 (48.6%)	1.000
BMI (kg/m^2)	26.8 [24.0; 30.0]	26.8 [24.0; 29.6]	27.1 [24.2; 31.6]	0.755
BSA (m^2)	1.85 [1.68; 2.01]	1.86 [1.67; 2.01]	1.82 [1.70; 2.03]	0.958
NYHA‐Class:				0.090
1	4 (2.26%)	3 (2.11%)	1 (2.86%)	
2	38 (21.5%)	27 (19.0%)	11 (31.4%)	
3	107 (60.5%)	92 (64.8%)	15 (42.9%)	
4	28 (15.8%)	20 (14.1%)	8 (22.9%)	
NYHA > 2	135 (76.3%)	112 (78.9%)	23 (65.7%)	0.156
Prior stroke	16 (8.89%)	11 (7.59%)	5 (14.3%)	0.203
Prior TIA	9 (5.00%)	6 (4.14%)	3 (8.57%)	0.380
Prior MI	17 (9.44%)	13 (8.97%)	4 (11.4%)	0.747
Arterial hypertension	169 (93.9%)	136 (93.8%)	33 (94.3%)	1.000
Insulin‐dependent diabetes mellitus	20 (11.1%)	17 (11.7%)	3 (8.57%)	0.769
Prior CABG	53 (29.6%)	46 (31.9%)	7 (20.0%)	0.237
Permanent pacemaker	37 (20.6%)	31 (21.4%)	6 (17.1%)	0.746
Rhythm				0.676
Sinus rhythm	96 (64.9%)	74 (62.2%)	22 (75.9%)	
Atrial fibrillations	36 (24.3%)	31 (26.1%)	5 (17.2%)	
Atrial flutter	5 (3.38%)	5 (4.20%)	0 (0.00%)	
Pacemaker	9 (6.08%)	7 (5.88%)	2 (6.90%)	
Other	2 (1.35%)	2 (1.68%)	0 (0.00%)	
Aortic valve area (VTI)	0.80 [0.70; 1.00]	0.80 [0.70; 1.00]	0.70 [0.60; 0.88]	0.101
Aortic stenosis > mild	154 (91.7%)	125 (92.6%)	29 (87.9%)	0.479
AV mean pressure gradient	36.5 [26.0; 46.8]	35.0 [27.0; 45.0]	41.0 [23.0; 49.0]	0.432
AV peak pressure gradient	61.0 [44.0; 78.0]	60.0 [44.2; 76.0]	67.0 [43.0; 82.0]	0.364
Aortic regurgitation > mild	77 (44.3%)	62 (43.7%)	15 (46.9%)	0.894
Mitral regurgitation > mild	45 (25.1%)	34 (23.6%)	11 (31.4%)	0.460
Tricuspid regurgitation > mild	39 (21.8%)	31 (21.5%)	8 (22.9%)	1.000
AV prosthesis type:				0.498
Stented valve	164 (91.6%)	133 (92.4%)	31 (88.6%)	
Stentless valve	15 (8.38%)	11 (7.64%)	4 (11.4%)	
Prostheses at increased risk for CO	81 (45.3%)	59 (41.0%)	22 (62.9%)	0.032
AV prosthesis size (mm):				0.548
19	5 (3.07%)	3 (2.31%)	2 (6.06%)	
21	58 (35.6%)	48 (36.9%)	10 (30.3%)	
23	51 (31.3%)	39 (30.0%)	12 (36.4%)	
25	26 (16.0%)	22 (16.9%)	4 (12.1%)	
26	2 (1.23%)	1 (0.77%)	1 (3.03%)	
27	17 (10.4%)	13 (10.0%)	4 (12.1%)	
29	4 (2.45%)	4 (3.08%)	0 (0.00%)	
AV prosthesis size < = 23 mm	114 (69.9%)	90 (69.2%)	24 (72.7%)	0.86
Mode of degeneration:				0.233
insufficiency	21 (11.7%)	15 (10.3%)	6 (17.1%)	
stenosis	88 (48.9%)	75 (51.7%)	13 (37.1%)	
mixed	71 (39.4%)	55 (37.9%)	16 (45.7%)	

Abbreviations: AV, aortic valve; BMI, body mass index; BSA, body surface area; CABG, coronary artery bypass grafting; CO, coronary obstruction; MI, myocardial infarction; NYHA, New York Heart Association; TIA, transitory ischemic attack; VTI, velocity time integral.

**Table 2:** CT‐based characteristics.

	All *N* = 180	Aligned *N* = 145	Misaligned *N* = 35	*p* value
Time to CT (days)	5.00 [3.00; 33.0]	5.00 [3.00; 34.0]	5.50 [2.25; 30.0]	0.552
Annulus maximum diameter	21.4 [20.0; 23.7]	21.4 [20.0; 23.9]	21.0 [20.8; 23.2]	0.886
Annulus mean area	336 [290; 387]	335 [288; 388]	339 [300; 387]	0.726
Height LCA	9.00 [6.00; 12.0]	9.00 [6.60; 12.0]	8.90 [5.55; 11.0]	0.379
Height RCA	10.0 [7.08; 14.0]	10.0 [7.00; 14.0]	10.5 [7.70; 13.8]	0.842
SoV width	30.4 [27.5; 33.7]	30.4 [27.8; 33.0]	30.5 [26.8; 35.0]	0.984
STJ width	30.0 [27.0; 32.8]	29.9 [27.2; 33.0]	30.0 [25.0; 32.0]	0.254
LCA eccentric	32 (18.5%)	29 (20.9%)	3 (8.8%)	0.169
RCA eccentric	54 (31.4%)	41 (29.7%)	13 (38.2%)	0.451

Abbreviations: CT, computed tomography; LCA, left coronary artery; RCA, right coronary artery; SoV, sinus of valsalva; STJ, Sinotubular junction.

### 3.3 Procedural Characteristics

Procedural approach for ViV‐TAVI was transfemoral in all but 2 cases, which were trans‐subclavian and resulted in CMA (*p* = 0.04; Table 3). Self‐expanding THV were used in 49.2% of the cases, without differences regarding the incidence of CMA. Of the 78 cases of self‐expanding THV used at Heart Center Leipzig, strategies to achieve commissural alignment were utilized in 56 cases (78%). Commissural alignment was confirmed in 63 cases (80.8%), with a greater incidence following intentional alignment (87.5% vs. 63.6%, *p* = 0.03). Strategies to prevent CO (i.e., leaflet laceration or pre‐emptive stenting) were applied in 56 cases (31.5%), 85.7% of which for prostheses at increased risk of CO. Both left main and right coronary eccentricity was more common in misaligned versus aligned prostheses (82.8% vs. 50.0% and 82.1% vs. 50.8%, *p* = 0.003 and 0.005, respectively).

**Table 3:** Procedural characteristics.

	All *N* = 180	Aligned *N* = 145	Misaligned *N* = 35	*p* value
ViV approach				0.037
Transfemoral	178 (98.9%)	145 (100%)	33 (94.3%)	
trans‐subclavian	2 (1.11%)	0 (0.00%)	2 (5.71%)	
ViV model				0.387
Sapien 3/Ultra	89 (49.7%)	69 (47.9%)	20 (57.1%)	
Sapien	2 (1.12%)	2 (1.39%)	0 (0.00%)	
Evolut Pro	3 (1.68%)	3 (2.08%)	0 (0.00%)	
Evolut R	82 (45.8%)	68 (47.2%)	14 (40.0%)	
Evolut Fx	2 (1.12%)	2 (1.39%)	0 (0.00%)	
Other	1 (0.56%)	0 (0.00%)	1 (2.86%)	
ViV model type				0.520
Balloon‐expandable	91 (50.8%)	71 (49.3%)	20 (57.1%)	
Self‐expanding	88 (49.2%)	73 (50.7%)	15 (42.9%)	
ViV size				0.039
20	11 (6.15%)	6 (4.17%)	5 (14.3%)	
23	99 (55.3%)	84 (58.3%)	15 (42.9%)	
26	54 (30.2%)	42 (29.2%)	12 (34.3%)	
27	1 (0.56%)	0 (0.00%)	1 (2.86%)	
29	14 (7.82%)	12 (8.33%)	2 (5.71%)	
ViV size ≤ 23 mm	110 (61.5%)	90 (62.5%)	20 (57.1%)	0.696
Cerebral protection	126 (70.4%)	99 (68.8%)	27 (77.1%)	0.442
Measures to prevent CO	57 (31.8%)	45 (31.2%)	12 (34.3%)	0.886
Pre‐emptive stenting	6 (3.33%)	4 (2.76%)	2 (5.71%)	0.331
BASILICA	51 (28.5%)	41 (28.5%)	10 (28.6%)	1.000
Leaflet modified				0.821
None	76 (59.4%)	62 (60.2%)	14 (56.0%)	
LCC	47 (36.7%)	37 (35.9%)	10 (40.0%)	
RCC	1 (0.78%)	1 (0.97%)	0 (0.00%)	
Both (Dual‐BASILICA)	4 (3.12%)	3 (2.91%)	1 (4.00%)	
Predilatation	22 (12.3%)	16 (11.1%)	6 (17.1%)	0.388
Postdilatation	77 (43.0%)	67 (46.5%)	10 (28.6%)	0.083
Fluoroscopy time	23.2 [16.0; 36.0]	23.0 [16.0; 33.0]	31.1 [15.0; 43.5]	0.182
Angle Diff. RCA‐RCC	5.00 [−6.00; 27.0]	4.00 [−9.00; 12.0]	47.0 [39.5; 59.5]	< 0.001
Angle Diff. RCA‐LCC	5.00 [−6.23; 25.0]	2.00 [−11.00; 12.0]	53.0 [43.5; 67.0]	< 0.001
Angle Diff. RCA‐NCC	5.00 [−9.00; 22.2]	3.00 [−16.00; 10.0]	41.0 [33.0; 52.0]	< 0.001
Average Diff. Alignment (°)	5.00 [−7.00; 23.8]	2.53 [−11.77; 10.7]	45.7 [38.0; 57.8]	< 0.001
LCA eccecntric post ViV	87 (56.1%)	63 (50.8%)	24 (82.8%)	0.003
RCA eccentric post ViV	87 (56.5%)	64 (50.(%)	23 (82.1%)	0.005

Abbreviations: CO, coronary obstruction; LCC, left coronary cusp; NCC, non‐coronary cusp; RCA, right coronary artery; RCC, right coronary cusp; ViV, valve in valve.

VARC‐3‐based technical success was lower for cases of CMA (80% vs. 93.1%, *p* = 0.03), driven by 14 cases of CO (including 1 case of VARC‐3 type‐5 myocardial infarction) in patients with CMA (17.1% vs. 5.6%, *p* = 0.03). Of the 14 cases of CO, 13 (93%) occurred in surgical prostheses at increased risk of CO, and 11 cases (79%) occurred following implantation of balloon‐expandable prostheses (*p* = 0.03). Multivariable logistic regression revealed the variable of *prosthesis at increased risk of coronary obstruction* as an independent predictor of CO (Supporting Information S1: Tables 2, 3).

### 3.4 Hemodynamic and Clinical Outcome

**Figure 2:**
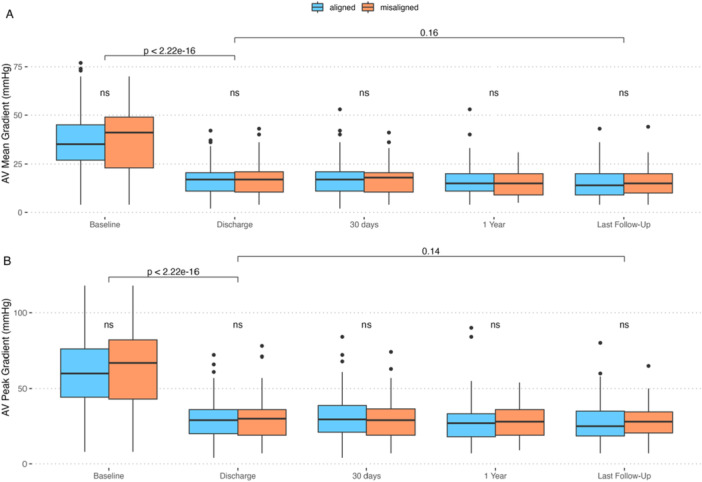
Graphs depicting mean (A) and peak (B) valve gradient by commissural alignment (orange = misaligned, blue = aligned). [Color figure can be viewed at wileyonlinelibrary.com]

**Figure 3:**
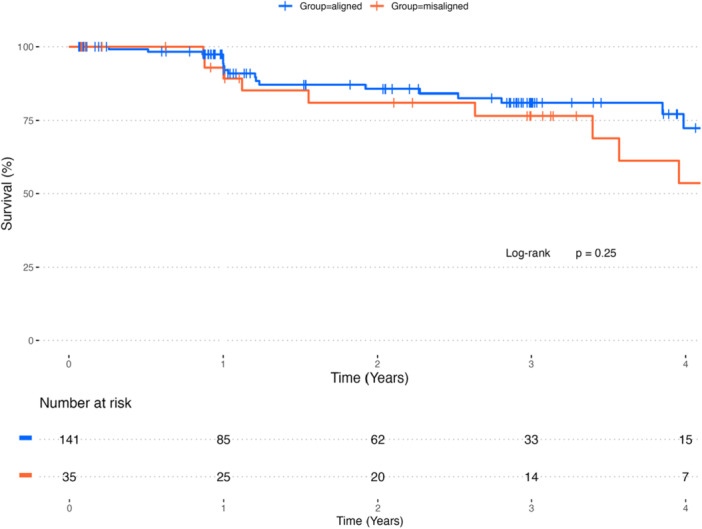
Curves depict Kaplan–Meier time‐to‐event rates for all‐cause mortality by commissural alignment (orange = misaligned, blue = aligned). [Color figure can be viewed at wileyonlinelibrary.com]

Periprocedural survival was excellent, with no operative or hospital mortality (Table 4). VARC‐3‐based device success was 62.9%, driven by the incidence of residual mean valve gradient (≥ 20 mmHg) of 35.4%, without differences between groups. Mean aortic valve gradient at discharge was 17 mmHg (IQR 11.0; 21.0 mmHg), comparable between groups, and remained unchanged until last follow‐up (Figure 2). The fraction of prosthesis regurgitation > trace based on discharge echocardiography was 3.4%, comparable between groups, and remained unchanged until the last follow‐up. The rate of HALT was 31.7% and comparable between groups. Midterm survival after a mean of 747 days was 84.6% and was not different between groups (log‐rank *p* = 0.25) (Figure 3).

**Table 4:** Clinical outcomes.

	All *N* = 180	Aligned *N* = 145	Misaligned *N* = 35	*p* value
Technical success	162 (90.5%)	134 (93.1%)	28 (80.0%)	0.027
Conversion to surgery	1 (0.56%)	0 (0.00%)	1 (2.86%)	0.196
Stroke	1 (0.56%)	1 (0.69%)	0 (0.00%)	1.000
Coronary obstruction	14 (7.82%)	8 (5.56%)	6 (17.1%)	0.033
Vascular/access‐related complication^a^				1.000
No	161 (89.9%)	129 (89.6%)	32 (91.4%)	
Major	1 (0.56%)	1 (0.69%)	0 (0.00%)	
Minor	17 (9.50%)	14 (9.72%)	3 (8.57%)	
Bleeding^a^				0.503
No	166 (92.7%)	135 (93.8%)	31 (88.6%)	
Type 1	11 (6.15%)	7 (4.86%)	4 (11.4%)	
Type 2	1 (0.56%)	1 (0.69%)	0 (0.00%)	
Type 3	1 (0.56%)	1 (0.69%)	0 (0.00%)	
Acute kidney injury^a^				0.215
No	174 (97.2%)	140 (97.2%)	34 (97.1%)	
Stage 1	4 (2.23%)	4 (2.78%)	0 (0.00%)	
Stage 2	1 (0.56%)	0 (0.00%)	1 (2.86%)	
Pacemaker implantation	8 (4.47%)	8 (5.56%)	0 (0.00%)	0.358
HALT	57 (31.7%)	48 (33.1%)	9 (25.7%)	0.522
Anticoagulation	84 (47.5%)	69 (48.6%)	15 (42.9%)	0.675
AR > trace	10 (5.56%)	8 (5.52%)	2 (5.71%)	1.000
PVL > trace	30 (16.7%)	25 (17.2%)	5 (14.3%)	0.866
Residual gradient (≥ 20 mmHg)	62 (35.4%)	48 (34.3%)	14 (40.0%)	0.664
PPM				0.904
Absent	55 (37.2%)	45 (36.9%)	10 (38.5%)	
Moderate	51 (34.5%)	43 (35.2%)	8 (30.8%)	
Severe	42 (28.4%)	34 (27.9%)	8 (30.8%)	
Device success	104 (59.4%)	85 (60.7%)	19 (54.3%)	0.617

Abbreviations: AR, aortic regurgitation; HALT, hypoattenuated leaflet thickening; PPM, patient‐prosthesis mismatch; PVL, paravalvular leak.

a Based on VARC‐3 definitions.

## 4 Discussion

This study analyzed the impact of CMA on clinical and hemodynamic outcomes in patients undergoing aortic ViV‐TAVI for failed surgical aortic bioprostheses. The main findings can be summarized as follows: (1) CMA occurred in approximately 20% of the cases, with a greater incidence in surgical valves at increased risk of CO; (2) the type of THV (i.e., self‐ vs. balloon‐expandable) did not affect the occurrence of CMA; (3) technical success was lower in cases of CMA, driven by increased rates of CO; (4) clinical and hemodynamic outcome was comparable in patients with and without CMA; (5) CMA did not affect the occurrence of HALT. Central Illustration 1

**Central Illustration 1:**
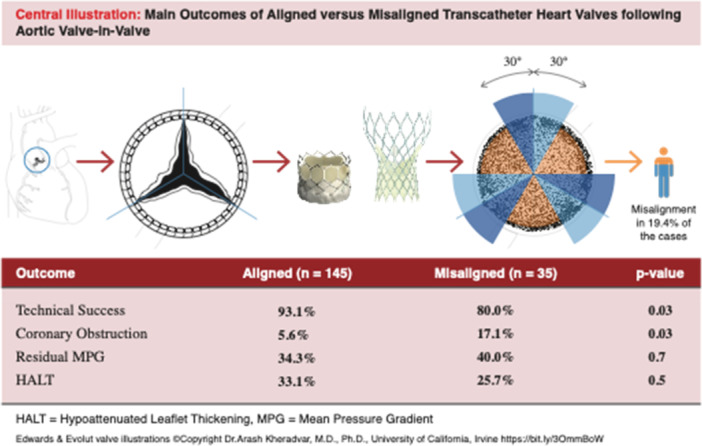
Showing the main outcomes of aligned versus misaligned transcatheter heart valves following aortic valve‐in‐valve. [Color figure can be viewed at wileyonlinelibrary.com]

To the best of our knowledge, the present study is the first to analyze the impact of CMA for ViV‐TAVI in a clinical cohort [16]. The lack of data likely results from the fact that ViV‐TAVI accounts for only 5% of all TAVI procedures, and post‐ViV CT scans are only performed for selected cases [12]. Of note, the indication for post‐procedural CT acquisition differed between the 2 centers providing data to this analysis, including clinical indications at Heart Center Leipzig, and a combination of both clinical and regulatory reasons at Cedars‐Sinai Smidt Heart Institute. The underlying data therefore, includes a selected sample in which post‐ViV CT scanning was performed, and results have to be interpreted accordingly.

We found that CMA occurred in approximately 20% of the cases, which is less frequent compared to native TAVI procedures, in which prostheses align randomly for cases in which strategies to achieve alignment are not utilized [10]. The low incidence of misalignment in the current study was somewhat surprising, in particular since a balloon‐expandable platform was used in approximately half the cases. Intentional alignment cannot be performed for the balloon‐expandable valves included in this analysis. Thus, in vitro bench testing may be required to potentially explain this observation. Regarding the decision about which THV platform is chosen for a ViV‐TAVI case, our data suggests that balloon‐expandable THV may be comparable in terms of the incidence of CMA following ViV‐TAVI.

### 4.1 CO

In our cohort, CO occurred in 14 cases (7.8%), which is more frequent compared to data from the Valve‐in‐Valve International Data (VIVID) Registry, in which CO occurred in 2.3% of the cases [6]. The fraction of prostheses at increased risk of CO in our sample, however, was 10% higher than the report by Ribeiro and colleagues [6]. In addition, coronary protection was performed frequently in our cohort (57 cases, 31.8%), reflecting a selection bias for individuals at increased risk of CO. It remains unclear why ViV‐TAVI for prostheses at increased risk of CO was more likely to result in CMA, which ultimately led to impaired VARC‐3‐based technical success for these cases. One possible explanation could be that the fluoroscopic properties of both the Mitroflow and Trifecta valves make commissural alignment during ViV‐TAVI more difficult, and stentless valves are even more problematic due to the complete absence of fluoroscopic markers. This could result in a higher risk of CO for these prostheses. The fact that multivariable regression revealed the type of surgical prosthesis as the only predictor of CO may support this assumption, although predicting a binary outcome (i.e., occurrence of CO) using correlating binary variables (i.e., CMA and type of prosthesis) is inconclusive.

On the other hand, our data suggest a possible association between CMA and CO. From a mechanistic perspective, it appears reasonable that techniques such as leaflet laceration could result in impaired coronary flow in cases of CMA, as the effect of leaflet laceration would then be neutralized by the covered commissural posts. However, BASILICA was used on both groups at a similar rate, without differences regarding the rate of adverse effects. Furthermore, due to the low rate of leaflet laceration in ViV‐cases with balloon‐expandable THVs in our cohort, in which CO occurred frequently, our data may suffer from a lack of power to clearly demonstrate an association between CMA and CO in this context. Thus, further study is required to test a potential interaction.

### 4.2 Hemodynamic Outcome

For native TAVI procedures, CMA has been reported to be associated with impaired hemodynamic outcome [9, 10]. Data on the impact of commissural alignment on hemodynamic outcome following aortic ViV‐TAVI, however, is lacking. Our data suggest that the rotational orientation of a THV within a deteriorated surgical aortic bioprosthesis does not affect hemodynamic outcome. Mean pressure gradients were reduced significantly following ViV‐TAVI, and remained stable until the last follow‐up, without differences between groups. In our dataset, the rates of elevated residual mean gradient (32.6%) and severe PPM (28.4%) were comparable to other ViV‐studies [17, 18]. Our data did not find an association of misalignment with paravalvular leakage or relative gradient increase. This can be explained by the technical differences between native and ViV‐TAVI, where in the latter, the circular bioprosthetic sewing ring serves as the anchor for the THV, which determines in large part the resulting valve orifice area and reduces the risk of paravalvular leakages, regardless of the rotational orientation of the THV.

### 4.3 Valve Thrombosis

Prosthetic leaflet thrombosis occurs more frequently in transcatheter compared to surgical aortic bioprostheses, with an incidence of subclinical valve thrombosis ranging between 13% and 32% following native TAVI [19, 20]. Predictors of leaflet thrombosis include balloon‐expandable prostheses and ViV procedures, in which the incidence of clinical valve thrombosis has been reported to be 7.3% [7, 21]. In our data, zero cases of clinical valve thrombosis were found, which is most likely due to the short time between ViV‐TAVI and post‐procedure CT‐scans (median time 5 days). In contrast, Abdel‐Wahab and colleagues reported that cases of clinical valve thrombosis were diagnosed at a median time of 101 days (IQR, 21–226) after the ViV‐procedure [7]. The incidence of HALT in our cohort was 31.7%, which is comparable to numbers reported following native TAVI. The rotational orientation did not seem to affect the incidence of HALT.

### 4.4 Limitations

The current study has several limitations. First, the underlying dataset is based on only 25% of the ViV‐cases performed over the study period, in which post‐ViV CT imaging was available. Second, the indication for CT scanning differed between the two institutions and did not follow a prospective study protocol, resulting in selection bias. Third, this was a retrospective study, and errors in data documentation or collection cannot be excluded. Finally, albeit being the largest study on this topic thus far, the sample size remains limited, resulting in a lack of power.

## 5 Conclusions

This is the first report on the impact of CMA on clinical and hemodynamic outcomes following ViV‐TAVI for failed surgical valves. CMA occurs at lesser rates compared to native TAVI cohorts, and does not seem to impact clinical or hemodynamic outcome. CO, however, occurred more frequently based on this dataset, which is suffering from a selection bias, including a large fraction of surgical prostheses at increased risk of CO.

## 6 Impact on Daily Practice

CMA during native TAVI procedures has been associated with hemodynamic outcome and coronary access. However, clinical data on the impact of CMA following ViV‐TAVI is scarce. This study demonstrates that CMA during ViV‐TAVI is a less frequent phenomenon that does not seem to impact short‐ and mid‐term hemodynamic or clinical outcome. Our data suggests a potential association between CMA and CO, although this finding is preliminary and hypothesis‐generating, suffering from a significant selection bias (only 26% of all ViV‐patients in this cohort at post‐procedure CT); the retrospective design; and the high incidence of prosthesis at increased risk of CO in this sample. Nevertheless, the increased risk of CO may encourage aligning TAVI‐prostheses during ViV‐procedures as well, in particular for surgical valves at increased risk of this complication.

## Disclosure

Dr. Borger declares that his hospital receives speakers' honoraria and/or consulting fees on his behalf from Edwards Lifesciences, Medtronic, Abbott, and Artivion. Dr. Abdel‐Wahab declares that his hospital receives speaker's honoraria and/or consultancy fees on his behalf from Medtronic, Boston Scientific, and Edwards Lifesciences.

## Conflicts of Interest

The authors declare no conflicts of interest.

## Supporting information

Examples of coronary obstruction and HALT_MR.tiff.

**Table S1:** Baseline characteristics by center. **Table S2:** Univariate logistic regression of predictors of coronary obstruction. **Table S3:** Multivariable logistic regression predictors of coronary obstruction.
