# Supplementary figures and images for: Commissural Misalignment Following Valve‐in‐Valve Transcatheter Aortic Valve Implantation
Source: Catheter Cardiovasc Interv. 2026 Jan 7;107(4):1215–24. doi: 10.1002/ccd.70460 (PMC12953210; doi:10.1002/ccd.70460)

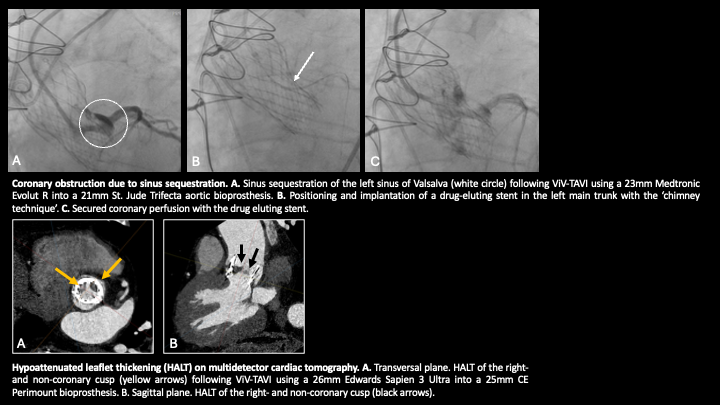

Supplement: Supplementary file 1 — Examples of coronary obstruction and HALT_MR.tiff. [file CCD-107-1215-s001.tiff]
