# Supplementary material for: Commissural Misalignment Following Valve‐in‐Valve Transcatheter Aortic Valve Implantation
Source: Catheter Cardiovasc Interv. 2026 Jan 7;107(4):1215–24. doi: 10.1002/ccd.70460 (PMC12953210; doi:10.1002/ccd.70460)
Supplement: Supplementary file 2 — Table S1: Baseline characteristics by center. Table S2: Univariate logistic regression of predictors of coronary obstruction. Table S3: Multivariable logistic regression predictors of coronary obstruction. [file CCD-107-1215-s002.docx]

**Supplemental Tables**

Table S1. Baseline characteristics by center

|  | Cedars N=78 | Leipzig N=102 | p-value |
| --- | --- | --- | --- |
| Age | 76.0 [68.0;83.0] | 79.0 [74.2;83.0] | 0.074 |
| Female: | 31 (39.7%) | 55 (53.9%) | 0.082 |
| BMI (kg/m^2) | 26.0 [24.0;29.3] | 27.4 [24.2;30.7] | 0.189 |
| BSA (m^2) | 1.91 [1.67;2.08] | 1.81 [1.68;1.96] | 0.130 |
| NYHA-Class: |  |  | 0.008 |
| 1 | 0 (0.00%) | 4 (3.92%) |  |
| 2 | 13 (17.3%) | 25 (24.5%) |  |
| 3 | 43 (57.3%) | 64 (62.7%) |  |
| 4 | 19 (25.3%) | 9 (8.82%) |  |
| NYHA>2 | 62 (82.7%) | 73 (71.6%) | 0.124 |
| Prior Stroke | 4 (5.13%) | 12 (11.8%) | 0.198 |
| Prior TIA | 8 (10.3%) | 1 (0.98%) | 0.011 |
| Prior MI | 4 (5.13%) | 13 (12.7%) | 0.140 |
| Arterial Hypertension | 72 (92.3%) | 97 (95.1%) | 0.535 |
| Insulin-Dependent Diabetes Mellitus | 3 (3.85%) | 17 (16.7%) | 0.013 |
| Prior CABG | 24 (31.2%) | 29 (28.4%) | 0.817 |
| Rhythm: |  |  | <0.001 |
| Sinus Rhythm | 20 (43.5%) | 76 (74.5%) |  |
| Atrial fibrillation | 14 (30.4%) | 22 (21.6%) |  |
| Atrial Flutter | 2 (4.35%) | 3 (2.94%) |  |
| Pacemaker | 8 (17.4%) | 1 (0.98%) |  |
| Other | 2 (4.35%) | 0 (0.00%) |  |
| Permanent Pacemaker | 17 (21.8%) | 20 (19.6%) | 0.862 |
| Aortic Valve Area (VTI) | 0.80 [0.70;1.15] | 0.75 [0.62;0.90] | 0.084 |
| Aortic Stenosis>mild | 64 (97.0%) | 90 (88.2%) | 0.086 |
| AV Mean Pressure Gradient | 37.0 [24.0;46.0] | 36.0 [28.5;48.5] | 0.292 |
| AV Peak Pressure Gradient | 63.5 [43.0;77.5] | 59.0 [47.0;78.0] | 0.702 |
| Aortic Regurgitation>mild | 33 (45.2%) | 44 (43.6%) | 0.952 |
| Mode of Degeneration: |  |  | <0.001 |
| Insufficiency | 10 (12.8%) | 11 (10.8%) |  |
| Stenosis | 63 (80.8%) | 25 (24.5%) |  |
| Mixed | 5 (6.41%) | 66 (64.7%) |  |
| Mitral Regurgitation>mild | 11 (14.1%) | 34 (33.7%) | 0.005 |
| Tricuspid Regurgitation>mild | 9 (11.5%) | 30 (29.7%) | 0.006 |
| AV Prosthesis Type: |  |  | 0.268 |
| Stented Valve | 74 (94.9%) | 90 (89.1%) |  |
| Stentless Valve | 4 (5.13%) | 11 (10.9%) |  |
| Prostheses at Increased Risk for CO | 25 (32.1%) | 56 (55.4%) | 0.003 |
| AV Prosthesis Size (mm): |  |  | 0.002 |
| 19 | 4 (6.35%) | 1 (1.00%) |  |
| 21 | 17 (27.0%) | 41 (41.0%) |  |
| 23 | 14 (22.2%) | 37 (37.0%) |  |
| 25 | 12 (19.0%) | 14 (14.0%) |  |
| 26 | 2 (3.17%) | 0 (0.00%) |  |
| 27 | 11 (17.5%) | 6 (6.00%) |  |
| 29 | 3 (4.76%) | 1 (1.00%) |  |
| AV Prosthesis Size <=23mm | 35 (55.6%) | 79 (79.0%) | 0.003 |

AV=Aortic valve, CABG=Coronary artery bypass grafting, CO=Coronary obstruction, MI=Myocardial infarction, NYHA=New York Heart Association, TIA=Transient ischemic attack, VTI = velocity time integral.

Table S2. Univariate logistic regression of predictors of coronary obstruction.

==============================================================

Dependent variable:

-----------------------------------------

Coronary Obstruction

----------------------------------------------------------------------------------------------------------

CMA 1.300** (0.130, 2.400)

High-risk prostheses 2.900*** (0.880, 5.000)

Self-expandable ViV-prostheses -1.400** (-2.700, -0.047)

STJ width -0.210*** (-0.350, -0.070)

Sinus of Valsalva width -0.170** (-0.320, -0.031)

==============================================================

Note: *p<0.1; **p<0.05; ***p<0.01

CMA = commissural misalignment, STJ = sinotubular junction, ViV = valve in valve.

Table S3. Multivariable logistic regression predictors of coronary obstruction.

==================================================================

Dependent variable:

---------------------------

Coronary Obstruction

-----------------------------------------------------------------------------------------------------------------

CMA 0.830 (-0.570, 2.200)

High-risk prostheses 3.500*** (1.200, 5.800)

Self-expandable ViV-prostheses -0.960 (-3.100, 1.100)

STJ width 0.054 (-0.170, 0.270)

Sinus of Valsalva width -0.120 (-0.320, 0.078)

Constant -2.400 (-8.700, 4.000)

------------------------------------------------------------------

Observations 173

Log likelihood -33.000

Akaike inf. crit. 79.000

==================================================================

Note: *p<0.1; **p<0.05; ***p<0.01

CMA = commissural misalignment, STJ = sinotubular junction, ViV = valve in valve.
